# Fibromuscular Dysplasia As a Rare Cause of Ileus and Aneurysm in Childhood

**DOI:** 10.1097/PG9.0000000000000045

**Published:** 2021-01-13

**Authors:** Nóra Judit Béres, Judit Szentannay, Attila Kálmán, Tímea Seszták, Ildikó Várkonyi, Judit Halász, Katalin Eszter Müller, György Balázs, Áron Cseh, Antal Dezsőfi

**Affiliations:** From the * First Department of Pediatrics, Semmelweis University, Budapest, Hungary; † Second Department of Pathology, Semmelweis University, Budapest, Hungary; ‡ Pediatric Institute-Clinic of the University of Debrecen, Debrecen, Hungary; § Heim Pál Children’s Hospital, Budapest, Hungary.

**Keywords:** aneurysm, fibromuscular dysplasia, ileus

**FIGURE 1.**
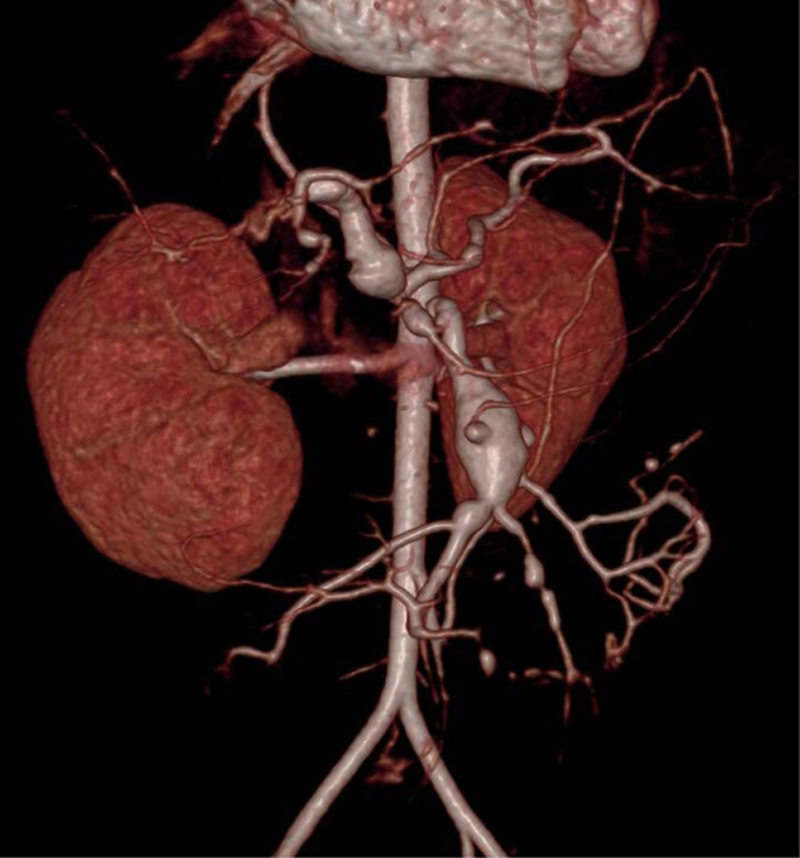
Three-dimensional computed tomography angiography, with fusiform aneurysms of superior mesenteric artery and hepatic artery.

A 21-month-old malnourished girl presented with abdominal pain and distension, vomiting, and radiological signs of ileus (dilated loops of small bowel proximal to the suspected obstruction, gasless abdomen). Barium enema ruled out Hirschsprung disease. Ultrasound revealed inflammatory changes in other small bowel loops. Because of worsening symptoms and persistent vomiting, laparoscopy was performed to evaluate for chronic intestinal pseudoobstruction, and an obstructed loop of jejunum adherent to the abdominal wall was identified. Adhesiolysis and resection revealed small bowel ulcerated mucosa and proliferated vascular intima. Inflammatory disorders of the vasa vasorum and other etiologies were not identified. Laboratory tests found no evidence for autoimmunity, celiac disease, cystic fibrosis, thrombophilia, or metabolic disorders. One week later, the child again developed ileus. Due to possible Crohn disease, exclusive enteral nutrition and corticosteroid treatment were introduced without success. Four months later, a second small bowel resection was required, and a similar obstructed, thickened loop was removed, with symptomatic relief. Abdominal sonography subsequently detected a fusiform aneurysmal dilation of the superior mesenteric and hepatic artery, confirmed by computed tomography angiography (Fig. 1). Magnetic resonance imaging and cardiac sonography revealed no other aneurysms. Histologic re-evaluation of material resected at both surgeries identified arterial changes of fibromuscular dysplasia (FMD).

FMD is a nonatherosclerotic, noninflammatory disease of the blood vessels that causes narrowing and “string-of-beads” feature of arteries, or even aneurysms or dissections. FMD most commonly affects the renal and carotid arteries, but it can occur in the mesenteric arteries, as in our case. The classification of FMD is according to the layer of the artery wall most involved: medial, intimal, and adventitial. In our case, we found intimal fibroplasia that affected the inner layer of the arterial wall (1). FMD is extremely rare in children (2–4). Symptoms of FMD are based on the location of the affected arteries. FMD has no definitive cure; treatment focuses on resolution of associated symptoms. Blood pressure control is a primary goal in renal involvement. Anticoagulation reduces the risk of stroke or blood clot formation. In our case, aspirin therapy was begun; however, asymptomatic hepatic artery thrombosis developed. Intervention, including angioplasty or surgical treatment, can be an option to improve blood flow or resolve aneurysms. In pediatric patients, it is important to improve lifestyle with diet, exercise, and smoking cessation. Follow-up must be life-long, with particular attention to renal artery involvement, hypertension, and clot formation (1).
